# Pain Intervention for people with Dementia in nursing homes (PID): study protocol for a quasi-experimental nurse intervention

**DOI:** 10.1186/s12904-017-0200-5

**Published:** 2017-04-21

**Authors:** Andrea Koppitz, Georg Bosshard, Geneviève Blanc, Hannele Hediger, Sheila Payne, Thomas Volken

**Affiliations:** 1 Zurich University of Applied Sciences, School of Health Professions, Institute of Nursing, Technikumstrasse 81, 8401 Winterthur, Switzerland; 2 University Hospital Zurich, Clinic for Geriatric Medicine and Centre on Aging and Mobility at the University of Zurich, Gloriastrasse 25, 8091 Zurich, Switzerland; 3 Lancaster University, International Observatory on End of Life Care, Division of Health Research, Faculty of Health and Medicine, Lancaster, LA1 4YG UK; 4 Zurich University of Applied Sciences, School of Health Professions, Institute of health science, Technikumstrasse 81, 8401 Winterthur, Switzerland

**Keywords:** Dementia, Pain, Intervention, Nursing home

## Abstract

**Background:**

It is estimated that 19 to 83% of people with dementia suffer from pain that is inadequately treated in the last months of life. A large number of healthcare workers who care for these people in nursing homes lack appropriate expertise and may therefore not always recognise, assess and treat pain in those with dementia who have complex problems on time, properly and efficiently. The aim of this intervention trial is to identify care needs of people with dementia suffering from pain living in a nursing home.

**Methods:**

A quasi-experimental nurse-led intervention trial based on a convenience sample of four nursing homes in the Swiss Canton of Zurich examines the effects on dementia patients (*n* = 411), the healthcare institution and the qualification level of the healthcare workers compared to historical controls, using an event analysis and a multilevel analysis. Healthcare workers will be individually trained how to assess, intervene and evaluate acute and chronic pain. There are three data-monitoring cycles (T0, T1, T2) and two intervention cycles (I1, I2) with a total study duration of 425 days. There is also a process evaluation based on Dobbins analyses that analyse in particular the potentials for change in clinical practice of change agents.

**Discussion:**

The aim of the intervention trial is to improve pain management strategies in older people with dementia in nursing homes. Clinically significant findings will be expected that will help reduce suffering in the sense of “total pain” for people with dementia. The joint intra- and interdisciplinary collaboration between practice and supply-oriented (nursing) research will have both a lasting effect on the efficiency measurement and provide scientifically sound results. Nursing homes can integrate the findings from the intervention trial into their internal quality control process. The potential for improvements can be directly influenced by the nursing home itself.

**Trial registration:**

Registration trial number: DRKS00009726 on DRKS, registered 10 January 2017, retrorespectively registered. Clearance certificate is available of the ethics committees of the canton of Thurgau, Switzerland, number: TG K201-02, and Zurich, Switzerland, number: ZH 01–2016.

## Background

Approximately 25% of people over the age of 85 years in Switzerland live in retirement and nursing homes. The average length of stay is 2.6 years [1]. More than half the people in nursing homes live with symptoms of dementia or have been diagnosed with dementia [2]. People with dementia show the characteristic symptom manifestation: 1) shortness of breath often combined with respiratory infections leading to pneumonia; 2) pain and 3) behavioural disorders, in particular agitation, restlessness and disorientation [3–13].

Of those people with dementia, 19–83% suffer from pain [7, 8, 10, 14–16] that is inadequately treated in the last months of life [17, 18]. The Pallhome study conducted in Switzerland identified pain and behavioural problems, as causing the greatest difficulties in controlling symptoms in dementia patients in nursing homes [19]. However, the fact that both symptoms can occur simultaneously makes it difficult to differentiate them [20, 21]. Zwakhalen and colleagues [14] also detected an increased risk of pain for people with multiple concurrent health problems and for people taking several analgesics. Both factors are true for people suffering from dementia in nursing homes. Untreated pain can lead to depression, sleep disorders, falls, malnutrition and deterioration in daily activities and physical functions [22]. In this context, various barriers to the treatment of pain in people with dementia have been identified [23–27] including: inadequate knowledge about the relationship between pain and behaviour; interpretation of behaviour; intensity of pain; pharmacological interventions and their side effects; non-pharmacological interventions; and interventions without prior clinical assessment by professionals. In addition, organisational frameworks such as the definition of responsibilities, the physicians’ claim that the patient had no pain and high staff turnover have been identified as barriers relevant to effective pain management [23, 25–28].

A daily diary for the detection and review of disease progression is considered an effective non-pharmacological intervention for pain. Primary nurses are present in nursing homes 24 h a day, 7 days a week, which means that they have detailed knowledge of the dementia patient, their needs and responses. They are therefore perfectly suited to assess the pain situation of older people in nursing homes. Equally important are individually tailored daily activities with one-to-one patient care, sufficient food and drink, breathing exercises and ergonomic adjustments in daily activities [24, 29]. The trial and error method can help to find out whether an analgesic helps or not when the presence of pain is unclear [24]. Case discussions are also used in everyday clinical practice. Their effectiveness has already been studied in the long-term care of dementia patients [30]. Hence they may be integrated as additional instruments in everyday life in nursing homes, but they are only conducted weekly or less often. Preliminary evidence shows that it is possible to provide care by qualified nursing staff that are continuously present, are suitably qualified, collaborate in interdisciplinary ways, and have target group-specific advanced knowledge. In addition, collaboration between nursing home physicians and advanced practice nurses in long-term care facilities have proven to be successful [31, 32].

As many as 52% of healthcare workers in Swiss nursing homes currently have no training or are employed at assistant level and have to cope with the complex needs of people with dementia [33]. Specific knowledge of pain assessment and management in everyday clinical situations in nursing homes with many differently qualified healthcare workers teams is lacking. Consequently this means that the older people suffering from highly complex health problems are cared for by a large number of staff with limited professional knowledge [2]. More specifically, this lack of competency may lead to delayed, improper and inefficient assessments and treatment of medical problems in complex situations such as pain in people with dementia. The largest group of Swiss healthcare workers in nursing homes, 60%, are healthcare assistants and only 27% nursing staff are qualified nurses [33]. It is therefore necessary to provide a communication and information environment that allows healthcare workers at assistant and secondary level to rapidly share observations, accurately and easily with healthcare professionals to initiate decision-making processes. A number of studies of quality of care in nursing home suggest that these measures are inadequately implemented in everyday clinical practice [2, 8, 34, 35]. The research question is therefore: does a nursing-led intervention reduce pain intensity and pain duration for dementia patients living in nursing homes?

Following this research question, the study aim is to identify care needs of people with dementia suffering from pain living in a nursing home. Primary outcomes are pain intensity and pain duration of people with dementia. Secondary outcomes include: differences in pain assessments between healthcare worker groups, behavioural problems, the use of psychopharmacological drugs, and the use of analgesics.

## Methods

We employ a quasi-experimental nursing-led intervention trial with a repeated measures design [36] based on a convenience sample of four nursing home clusters and a total sample of 411 dementia patients. All dementia patients in the comparison group will be obtained from the Zurich Life And Death with Advanced Dementia (ZULIDAD) Study [37]. The ZULIDAD data were collected from people with dementia (men and women), who are older than 70 years in one nursing home (*n* = 150), in 2015. Data between intervention sites and comparison site are similar because the same assessment instruments were used, based on an older person assessment instrument, and include pain duration, pain intensity, behavioural problems, differences of psychopharmacological drugs, and differences of analgesics. The comparison can be made with data from the first day and from the 49^th^ of each data collection phase (t0, t1, t2) and with a historical sample of pain duration and pain intensity from the last pain assessment documented in the older person Assessment Instrument Data (RAI) of each participant.

People with dementia in the intervention group will be are recruited in three healthcare institutions in the cantons of Thurgau and Zurich.

All healthcare workers from these three intervention institutions are included in the study, trained and coached in the provision of interventions. The following inclusion criteria apply to healthcare workers:

- must be at least 18 years old;
- must have been employed at least 12 months in the respective organisation;
- must work for at least 30% of a working week;
- be able to communicate in German.

The collection of data will take place in three nursing homes in the Canton of Thurgau and in the Canton of Zurich. For the comparison group, data from the ZULIDAD study [37] will be used.

**Fig. 1:**
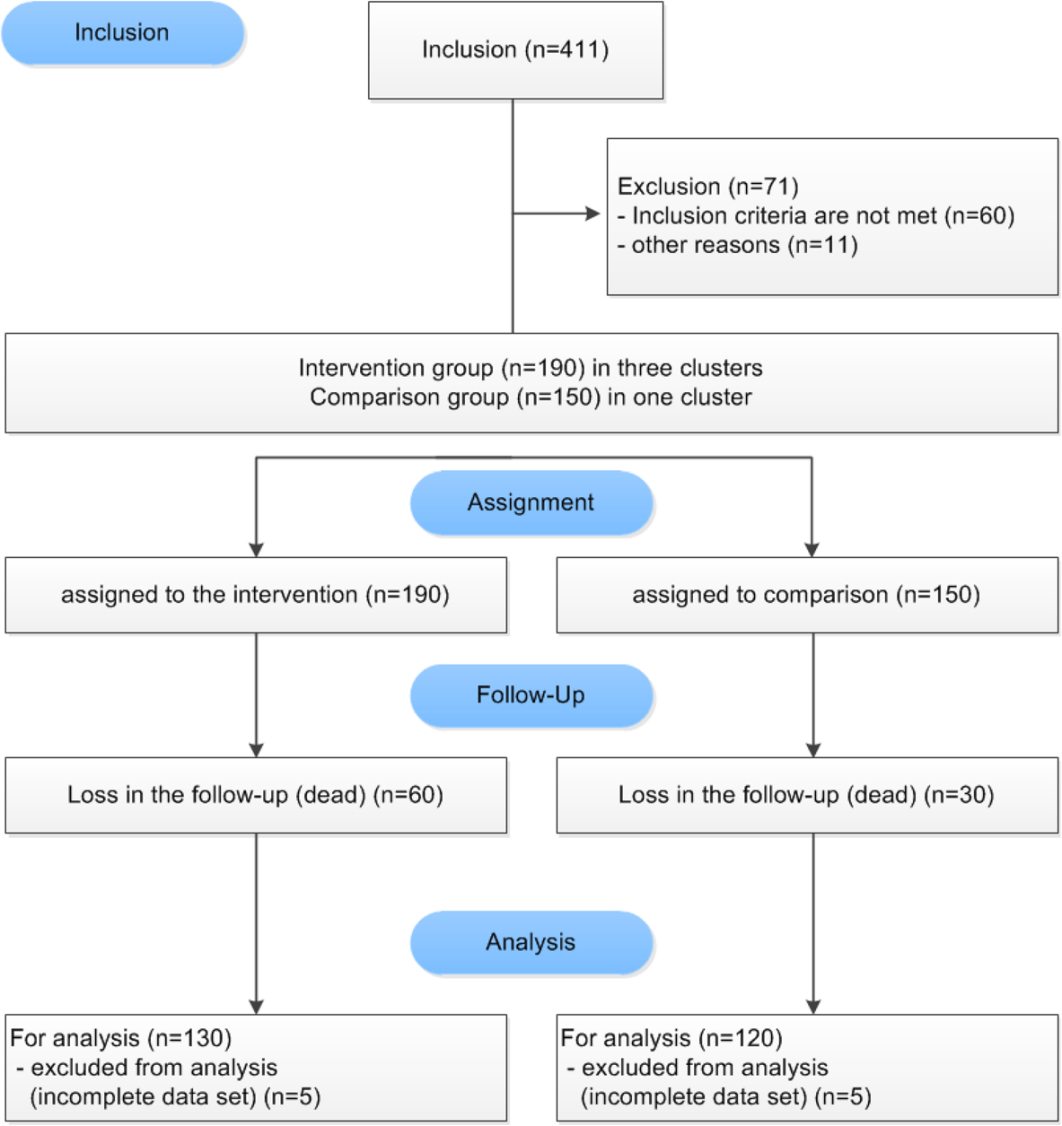
Recruitment

All older people are screened for eligibility to participate in the study. Recruitment is described in Fig. 1. Only older people with documented nursing-initial-assessment symptoms of dementia are included in this intervention trial by author no. 3.

**Fig. 2:**
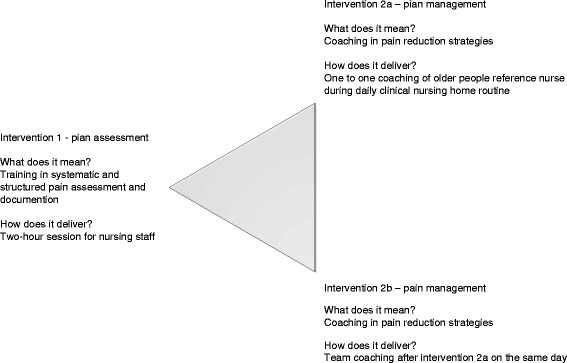
Intervention 1 and 2

The intervention, see Fig. 2, is delivered by a nurse with a Master of Science in nursing degree who has been working as an registered advanced practice nurse [31, 38]. At weekly 1 hour meetings, of the Research Intervention Team with the specialist in internal medicine who focuses on geriatrics and the principal investigator, the intervention nurse reflects on her training, consulting and support experience. The intervention consists of two parts, which are described in detail below:

Intervention 1 – pain assessment

Aim: to reinforce the systematic pain assessment of healthcare workers.

1. Encourage training of healthcare workers on pain assessment using the BESD instrument (assessment of pain in dementia of the German Pain Society) by the intervention nurse [39–41].
2. Individual coaching of healthcare workers (2× per healthcare worker at bi-weekly intervals) on the correct use of the BESD in everyday clinical life in nursing homes and a systematic and structured communication among healthcare workers and between healthcare workers and physicians based on ISBAR [42] and SOAPIER [43]. ISBAR is clinical communication technique (ISBAR: identify, situation, background, assessment, recommendation). SOPIER is a problem-oriented technique whereby the nurse identifies and lists the patient’s health concerns (SOPIER: subjective data, objective data, assessment data, plan, intervention, evaluation, recommendation).

Intervention 2 – pain management

Aim: to reinforce the systemic pain management skills of healthcare workers including systematic documentation and information among nursing staff and general practitioners.

1. Provide individual support and advice to healthcare workers on how to develop a Pain Action Plan in order to implement assessment and management and to evaluate them for each person affected with dementia suffering from pain or suspected pain.
  Zwakhalen, Hof, Hamers [44] concluded that the presence of pain should be assumed at a BESD value of two or higher. However, even at values of zero point on the BESD scale the presence of pain cannot be ruled out with certainty only because the person in question shows no discernible pain behaviour. That is why a professional exchange of information among nurses is vital for different pain management strategies to be tested in case of suspected pain or pain following a “trial and error” approach in consultation with the attending physician, if necessary, and to assess the success or failure of these strategies. Accordingly, there is a need for systematic observation by staff members who will be specifically trained for this task in intervention 1.
2. Discussions with healthcare workers based in a hospital ward and the intervention nurse on issues in the implementation of individual Pain Action Plans and strategies. The older person’s reference nurse calls for a meeting with members of the nursing team, the attending physician and the intervention nurse when she has uncertainties, in the care of newly admitted older person, in case of suspected pain or in case of changes to the older person’s therapy.

Sustainability and veracity of the interventions are ensured by following the comprehensive evaluation process based on Dobbins [45] that consists of:

- assessment of the accuracy between the conducted intervention and the study protocol: 50% of the intervention protocols are tested for compliance with the study protocol based on selected pain management strategies and structured communication skills (ISBAR, SOPIER, prompt initiation of medical prescriptions, usage of non-analgesic pain management strategies) by descriptive analysis.
- evaluation of operating procedures that are associated with pain management
- questioning of acceptability of the interventions carried out for all health care workers with a scale from 0 (= no acceptance) to 10 (= fully accepted)
- compliance (adherence) of interventions for all health care workers with a scale from 0 (= no adherence) to 10 (= complete adherence)
- assessment of the potential for change in clinical practice among healthcare workers with a scale from 0 (= no conversion potential) to 10 (= complete transformation possible)
- assessment of potential change in the management of pain among health care workers with a scale from 0 (= no change in potential) to 10 (= comprehensive amendment potential)

Pain as primary outcome is measured in all people with dementia in the comparison and intervention groups by means of:

- pain events per shift and per week;
- degree of pain intensity per work shift, per week day;
- pain-free intervals.

Secondary outcome variables will be measured by:

- frequency of behavioural problems (Neuropsychiatric Inventory NPI [46]);
- number/dosages of psychotropic drugs;
- Number/dosages of analgesics.

The documentation regarding the assessment of pain by nurses is always done in duplicate, by a health care worker on different education level. Tertiary outcome variables will be measured by:

- changes in the assessment of pain by healthcare worker on healthcare assistant level and
- changes between the two healthcare worker groups on healthcare assistant level and registered nurse level.

Hypotheses for primary outcomes are:

- H_a1_: There is a 20% reduction in pain events over time before and after the intervention and between intervention and comparison groups among nursing home older people.
- H_a2_: There is a 50% reduction in pain intensity over time before and after the intervention and between intervention and comparison groups among older people.
- H_a3_: There is a 20% reduction in the duration of pain free intervals over time before and after the intervention and between intervention and comparison groups among older people.

Furthermore the following hypotheses will be explored:

- There is a difference in the behavioural problems score (NPI) before and after the intervention and between intervention and comparison groups among older people.
- There is a difference in the psychopharmacological drug numbers/dosage before and after the intervention and between intervention and comparison groups among older people.
- There is a difference in the numbers/dosage of painkillers before and after the intervention and between intervention and comparison groups among older people.
- There is a difference in pain assessment over time before and after the intervention and between intervention and comparison groups among nursing home older people.
- There is still a difference in pain assessment by healthcare workers on assistant level over time before and after the intervention
- There is still a difference in pain assessment between healthcare workers on assistant level and registered nurses level over time before and after the intervention.

The sample size used in the trial is determined based on the requirements of event history analyses and multilevel analyses to test the mentioned hypotheses.

Event history analyses: The required number of events for a two-tailed test of individual coefficients within a Cox model with other covariates has been calculated based on the method of Hsieh and Lavori [41]. A total of *n* = 179 people and 121 events are required for a power of 0.8 and a significance level of 0.05 based on an expected hazard ratio of 0.6 (SD 0.5), a probability of occurrence of 0.75 and a drop-out rate of 10%. Both the number of people and the number of events determined in the intended study protocol can be collected.

Multilevel analyses (linear mixed models): The required number of cases for basic evaluations using multilevel models/linear mixed models have been determined by the approximation method [47] - with equal power and significance level as in the event analyses. This approach recognises a random intercept model with covariates that only vary between older people (treatment). In order to carry out only two repeated measurements per person, approximately *n* = 47 persons are required using a regression coefficient of 1 and a standard deviation of 0.5.

**Fig. 3:**
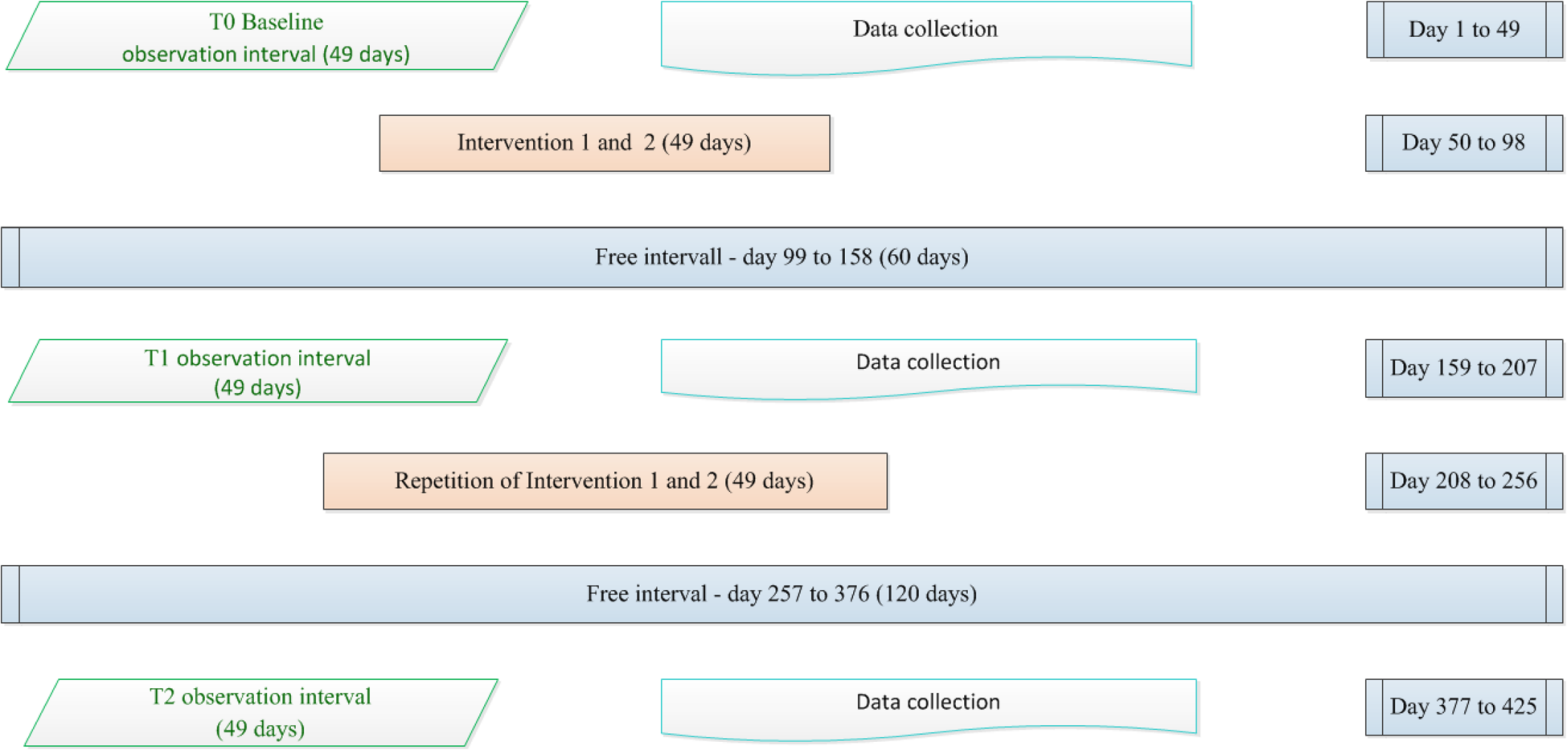
Time schedule

There are three data monitoring cycles (T0, T1, T2) and two intervention cycles (I1, I2) to measure the primary and secondary outcomes, see Fig. 3. Each data monitoring cycle extends over 7 weeks (=49 days) and includes seven different weekdays and at least three different work shifts. The data are extracted from the older people’ specific pain diaries (degree of pain intensity), which are kept separately from conventional nursing documentation. For the description of the sample, socio-demographic data on age and gender are collected from all participants. Additional data on qualifications and professional experience are collected from nursing staff, and care needs, dementia type and severity of dementia from people with dementia.

The actual data collection will be done by a study nurse with a BSc in Nursing. Potential confusions/problems with the raw data are discussed within the respective healthcare institution and with the leader of the study. Ambiguities with the procedure of data collection are discussed with the head of project management within the research team who also keeps a research diary. The study nurse transfers the anonymised data to the project management after the third measuring cycle. Two other study nurses enter the data into SPSS and carry out data cleansing before proceeding with the data analysis. The data of the comparison group are disclosed to the project management “PID” in encrypted form by the project manager “ZULIDAD”.

Data analysis will be performed using the IBM software programme SPSS® (version 24). The socio-demographic data are analysed descriptively (frequencies, central tendencies, standard deviations) and the outcome variables as follows:

- Pain intensity, duration and pain-free intervals are investigated in all dementia patients of the comparison and intervention group measuring the average duration of pain events per work shift (each work shift lasts 8.25 h) and per weekday, and the average degree of pain intensity per work shift, per work day as well as the duration of pain-free intervals with multilevel and events history analyses based on the data observation intervals T0, T1, T2.
- For the multilevel analyses (linear mixed models) over time, two levels are considered: the dementia patient and the healthcare institution. The event history analyses use cluster-adjusted Cox regressions. Furthermore, event history analyses will focus on evaluating the influence of the two nursing professional groups (at assistant/secondary and tertiary level), the pain pharmacotherapy (number and dosage), behavioural problems (NPI) and the psychopharmacotherapy (number and dosage).

A change in the assessment of pain by the healthcare workers is checked with interrater reliability tests. By means of the interrater reliability test, a potential variation between the two nursing groups on assistant/secondary and tertiary level is monitored.

To ensure the quality of data analyses, each data analysis step is independently checked by three different people.

Monitoring will by done by

- a trial master file which will be saved on a password-protected server with backup.
- All project steps are recorded in a diary and stored in the project folder. Project members will be instructed by the principal investigator. Their quality of work will be tested at the beginning of their activity.
- The data of the study participants are reviewed by external study nurses for completeness and anonymity and, if necessary, the contact person in the respective retirement and nursing home is consulted. This process is designed to protect the participants by ensuring that no personalised data leaves the nursing homes before it is entered into the statistics programme. The clinical trial meets the requirements of the Swiss Law on Human Research and adheres to the statutory provisions.

The intervention trial has been approved by the ethics committees of the canton of Thurgau (K2016/02) and Zurich (01–2016) with clearance certificates and will be conducted according to Good Clinical Practice ICH Expert Working Group (1996) [48], which is based on the Declaration of Helsinki (2013) [49]. The nurse-led intervention trial complies with the following basic ethical principles of research [50, 51]: respect of persons, beneficence and justice. The authors of the intervention trial declare no conflict of interest. The process evaluation, the intervention protocol and the results of the trial will be published.

## Discussion

The aim of the intervention trial is to improve pain management strategies in older people with dementia in nursing homes. We expect clinically significant findings that will help reduce suffering in the sense of “total pain” for people with dementia. The joint intra- and interdisciplinary collaboration between practice and supply-oriented (nursing) research will have both a lasting effect on the efficiency measurement and provide scientifically sound results. Nursing homes will be able to integrate the findings from the intervention trial into their internal quality comparison processes. The potential for improvements that can be directly influenced by the nursing home itself will be discussed. This means that the nursing homes can respond directly and immediately to deficits. Healthcare workers will be trained and the outcomes of the training will be measured by differences in pain management strategies by people with dementia. People with dementia are therefore protected from direct study interventions.

However, frustration and decreased motivation among healthcare workers could be possible due to intensive study training session during intervention 1 and 2. The study will measure these possible effects during the process evaluation.

Nevertheless, specific knowledge of assessing pain in the everyday clinical situation in many differently qualified healthcare worker teams in nursing homes is lacking. The present intervention trial therefore leads to a broadening of perspectives in palliative care and fills an important gap in the palliative care of people with dementia in nursing homes.
